# Supplementary material for: Meta-analysis reveals negative but highly variable impacts of invasive alien species across terrestrial insect orders
Source: Nat Commun. 2026 Jan 15;17:296. doi: 10.1038/s41467-025-67925-9 (PMC12808215; doi:10.1038/s41467-025-67925-9)
Supplement: Supplementary file 1 — Supplementary Information [file 41467_2025_67925_MOESM1_ESM.pdf]

Supplementary Information for

Meta-analysis reveals negative but highly variable impacts of invasive alien species across terrestrial insect orders.

Skinner *et al.*

This file includes:

Supplementary Figs. 1 to 5
Supplementary Tables 1 to 3
Supplementary Note 1: Meta-analysis protocol
Supplementary Note 2: Koricheva and Gurevitch (2014)[1] checklist
Supplementary Note 3: Citations for all studies included in the meta-analysis
Supplementary References

Other Supplementary Materials for this manuscript included as separate files:

Supplementary Data 1. Study screening outcomes and exclusion reasons

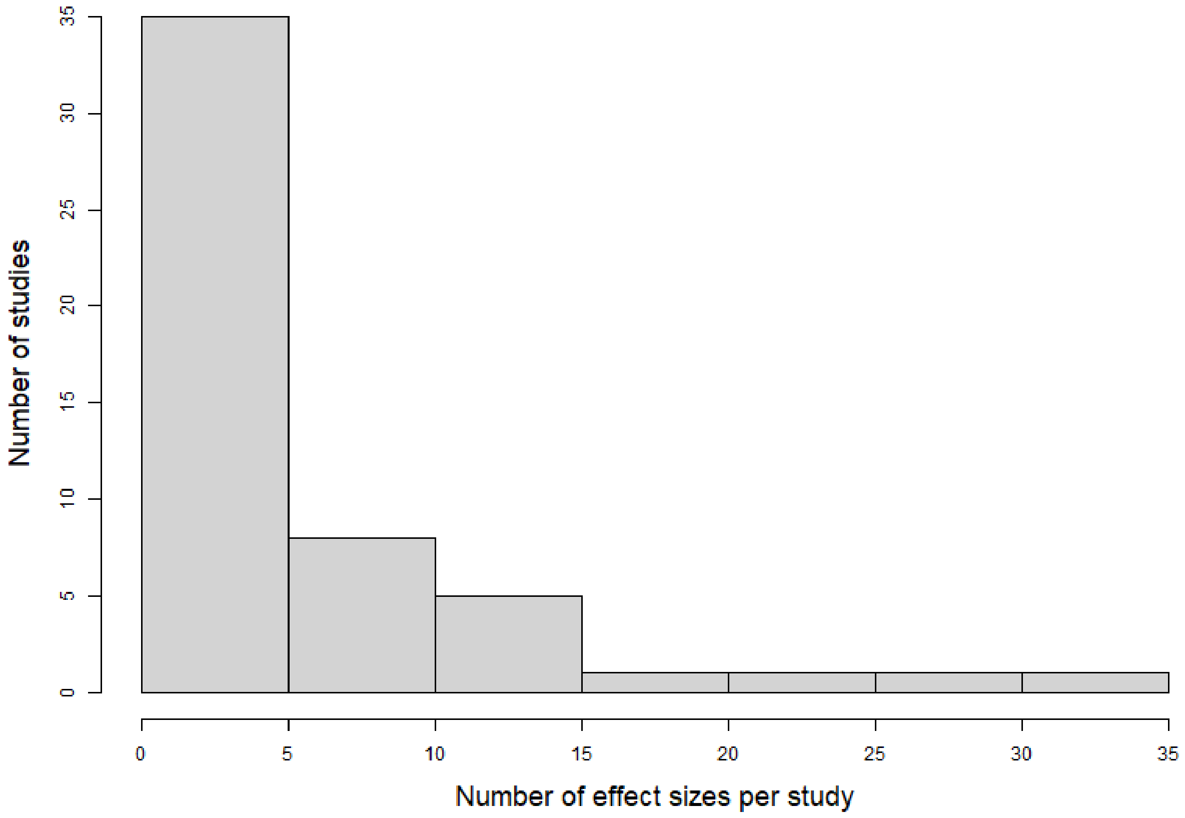

Supplementary Figure 1. Effect sizes per study. Distribution of effect sizes contributed per study in the meta-analysis. Each bar represents the number of studies contributing a specific range of effect sizes, with most studies contributing 5 effect sizes or fewer.

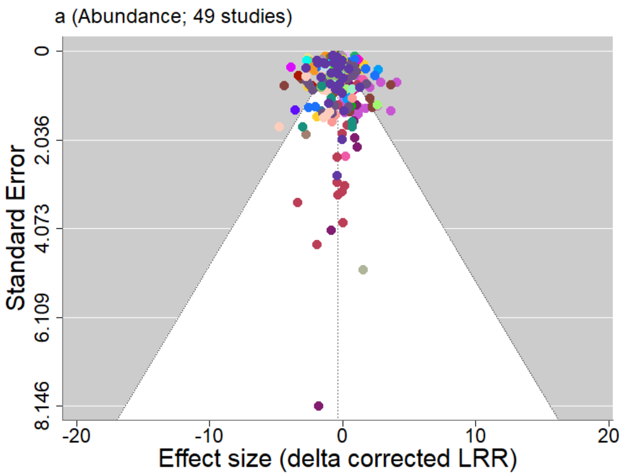

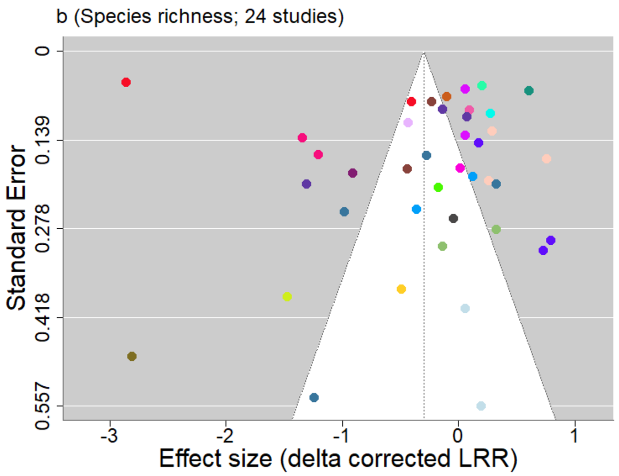

Supplementary Figure 2. Funnel plots used to assess publication bias. Effect sizes (delta corrected Log Response Ratio [LRR]) against the standard error describing the impact of invasive alien species presence on terrestrial insect (Coleoptera, Hemiptera, Hymenoptera, Orthoptera) (**a**) abundance and (**b**) species richness. Points are coloured by study.

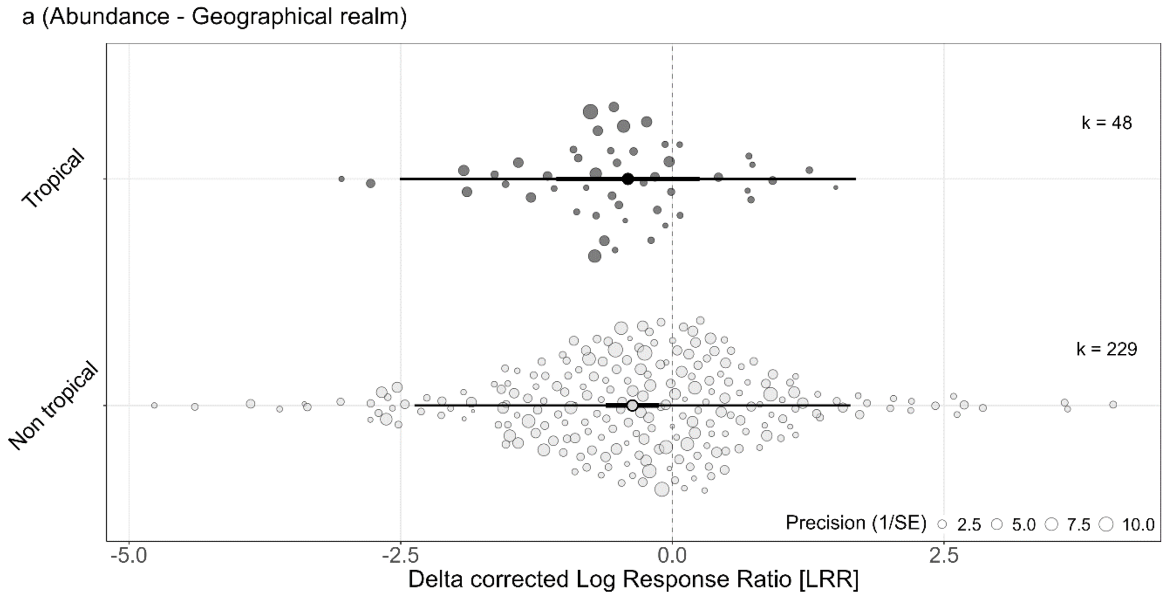
a (Abundance - Geographical realm)
Tropical
Non tropical
k = 48
k = 229
Precision (1/SE) 2.5 5.0 7.5 10.0
-5.0
-2.5
0.0
2.5
Delta corrected Log Response Ratio [LRR]

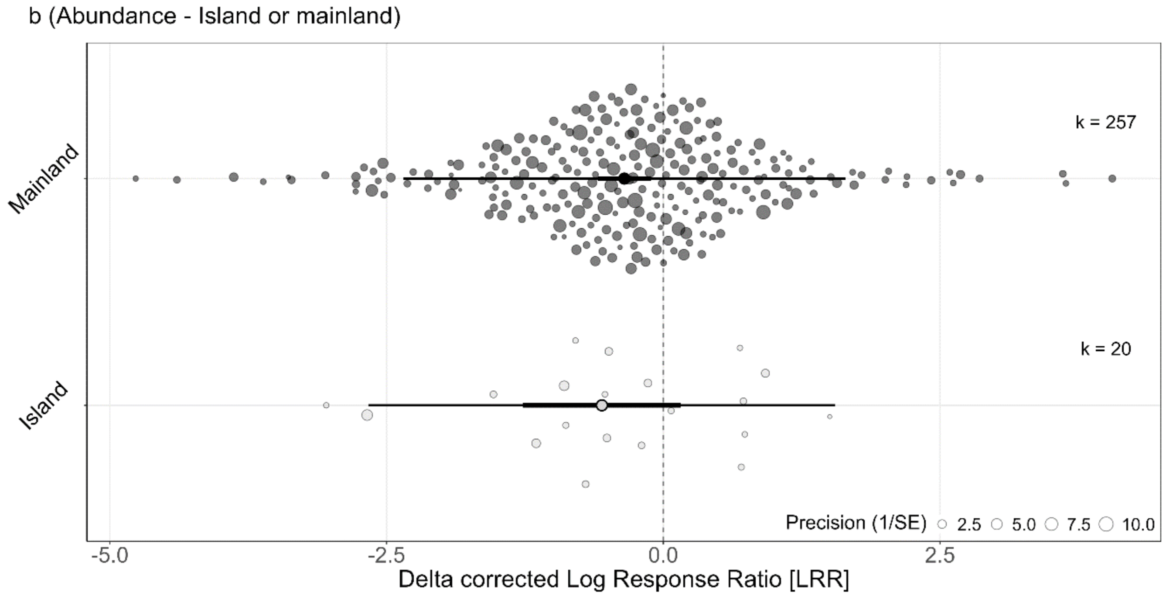
b (Abundance - Island or mainland)
Mainland
Island
k = 257
k = 20
Precision (1/SE) 2.5 5.0 7.5 10.0
-5.0
-2.5
0.0
2.5
Delta corrected Log Response Ratio [LRR]

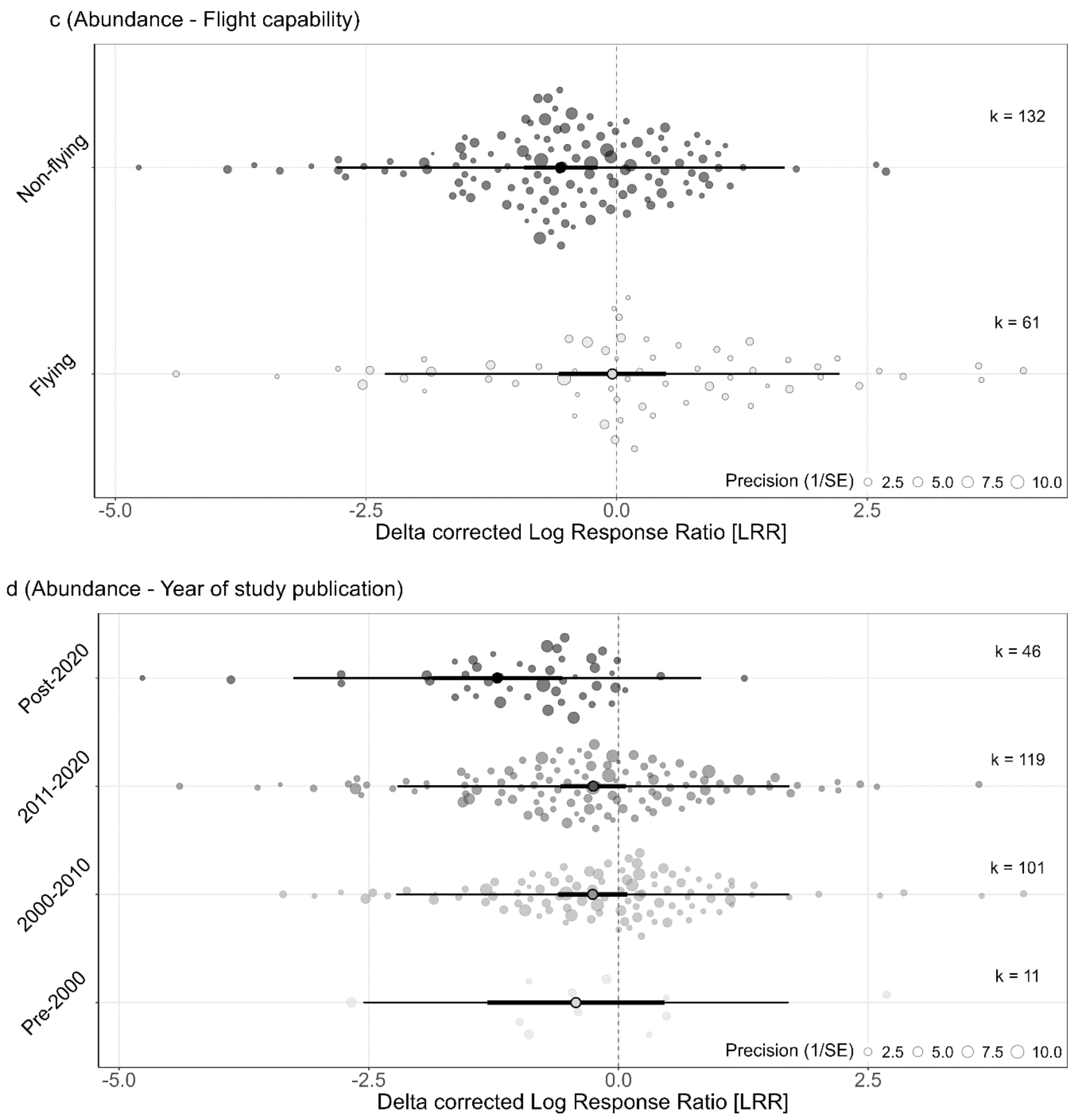

Supplementary Figure 3. Effect of invasive alien species on insect abundance, split by geographic realm, island or mainland, flight capability, and year of study publication. Model-derived response (delta corrected Log Response Ratio [LRR]) of insect abundance to invasive alien species presence split by (**a**) effect sizes originating from tropical (black) and non-tropical (grey) geographical realm; (**b**) effect sizes originating from mainlands (black) and islands smaller than 25,000 km^2 (grey); (**c**) effect sizes originating from focal insects with the ability to fly (grey) or not (black); (**d**) effect sizes originating from studies published pre-2000 (light grey), 2000-2010 (grey), 2010-2020 (dark grey), and post-2020 (black). Plots derived from results of single-moderator metafor[2] models run with abundance data with (**a**) geographic realm, (**b**) island or mainland, (**c**) flight capability, and (**d**) year of study publication as a moderator variable. The QM test for moderators showed none of these variables explained a significant proportion of variation in effect sizes. k represents the number of effect sizes for each grouping,

as indicated on the plot. For each grouping, the solid dot represents the model-derived overall estimated effect size (delta corrected LRR), with thick black bars indicating the 95% confidence intervals, and thinner black bars the prediction intervals. Effect sizes for each data point are represented by the translucent circles, with circle size representing its weighting in the model. The positioning of each circle on the y-axis is so all points can be seen (jittered).

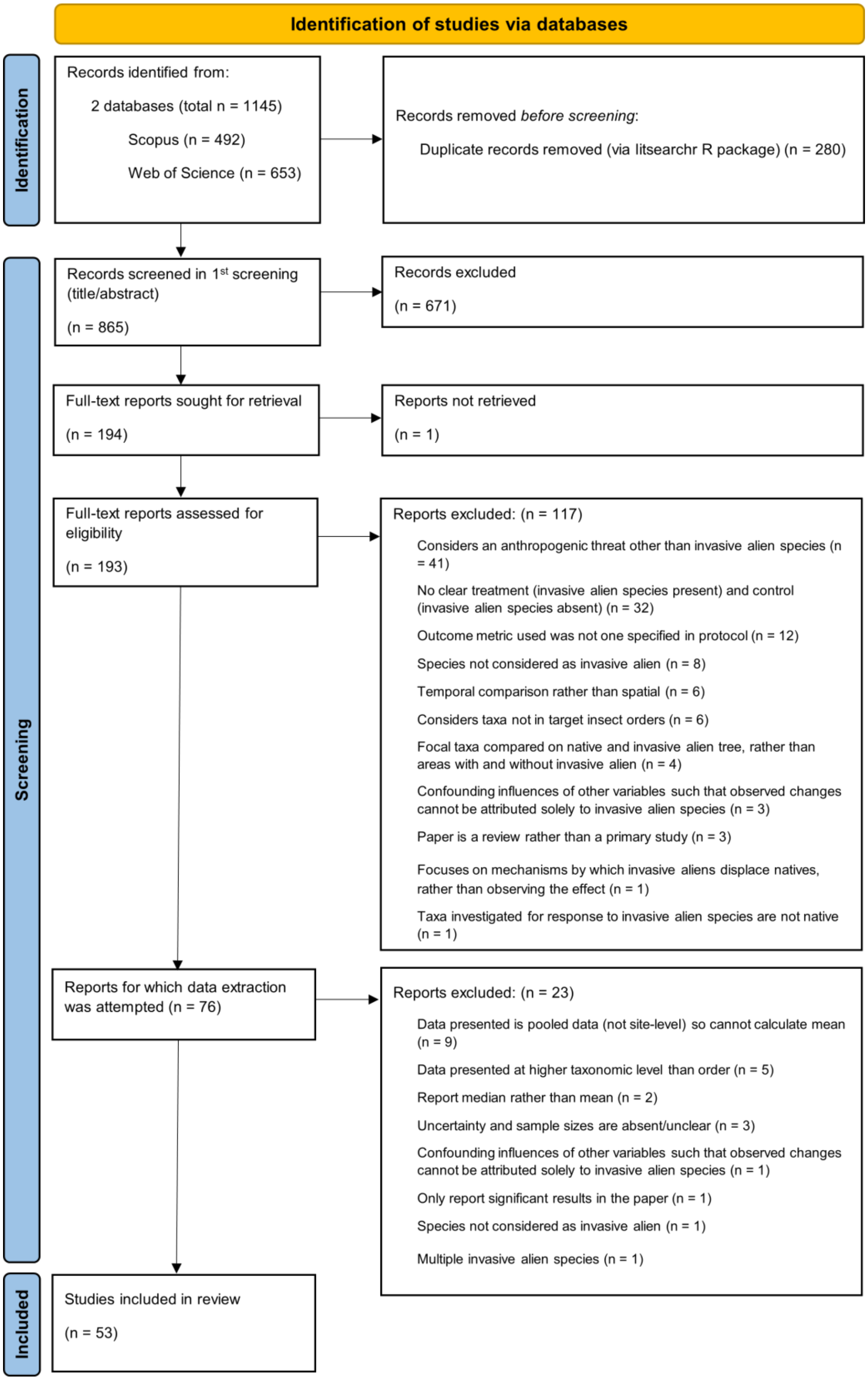

Supplementary Figure 4. PRISMA diagram. PRISMA diagram outlining the process of searching for and selecting studies to include in the meta-analysis. n represents the number of studies at each stage.

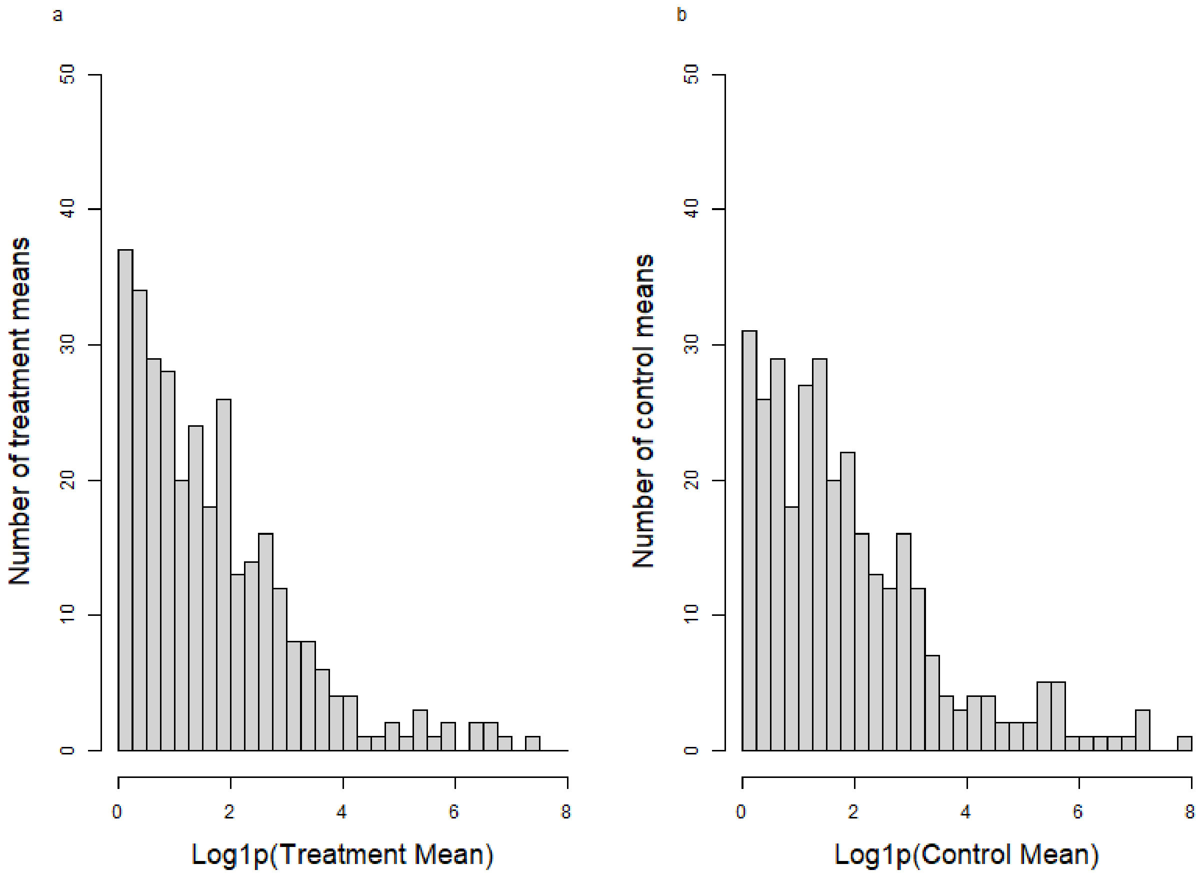

Supplementary Figure 5. Distribution of transformed mean biodiversity measures for treatment and control sites. Distribution of log(mean + 1) from (**a**) treatment (invasive alien species present) sites and (**b**) control (invasive alien species absent) sites. The x-axis shows log(mean + 1) values (computed using the base R function log1p) As a high proportion of our extracted mean biodiversity measures were close to zero, we applied a bias correction to our effect sizes and associated variances using the delta method[3].

Supplementary Table 1. Results of sensitivity tests. Results for the sensitivity tests conducted for the abundance and species richness models.

Biodiversity metric	Sensitivity test	Sample size	Estimate (95% confidence intervals)	Estimate in % change
**Abundance**	Swapping Log Response Ratio (LRR) for Hedge's g (standardised mean difference) as the effect size	277	-0.3423 (-0.5754, -0.1092)*	NA
	Cook's distance - excluding influential data points where their Cook's distance exceeded 4/N	269	-0.3526 (-0.5545, -0.1506)*	-29.71% (-42.57%, -13.98%)
	Excluding data collected with aquatic sampling techniques	242	-0.3567 (-0.5982, -0.1153)*	-30.00% (-45.02%, -10.89%)
	Geary's test - excluding data where the small sample corrected standardized mean of either the treatment or control did not pass Geary's test	95	-0.3401 (-0.5930, -0.0872)*	-28.83% (-44.73%, -8.35%)
	Incorporating a phylogenetic correlation matrix as a random effect^	111	-0.3621 (-1.2246, 0.5005)	-30.38% (-70.61%, 64.95%)
**Species richness**	Swapping Log Response Ratio (LRR) for Hedge's g (standardised mean difference) as the effect size	40	-0.4003 (-1.0393, 0.2387)	NA
	Cook's distance – excluding influential data points where their Cook's distance exceeded 4/N	37	-0.1457 (-0.3619, 0.0704)	-13.56% (-30.36%, 7.3%)
	Excluding data collected with aquatic sampling techniques	38	-0.3198 (-0.6197, -0. 0198)*	-27.37% (-46.19%, -1.96%)
	Geary's test - excluding data where the small sample corrected standardized mean of either the treatment or control did not pass Geary's test	34	-0.1903 (-0.4688, 0.0882)	-17.33% (-37.42%, 9.22%)

*Significant (Confidence intervals do not overlap 0).
^A model incorporating a phylogenetic correlation matrix as a random effect had a greater AIC value (AIC = 385.8) than an equivalent model without (AIC = 384.7).

Supplementary Table 2. Results of the multi-moderator meta-regression model using the abundance dataset. Output of an abundance model including all moderators simultaneously (k = 193). Estimates represent the difference in effect size for each moderator level relative to the baseline (reference) level of each moderator, controlling for all other moderators in the model.

**Moderator**	**Estimate (95% confidence intervals)**	**Standard error**
Intercept	-0.0163 (-2.5838, 2.5512)	1.3100
Order: Hemiptera	-1.4891 (-2.2849, -0.6932)	0.4061
Order: Hymenoptera	-0.0046 (-0.6329, 0.6238)	0.3206
Order: Orthoptera	-1.3549 (-3.4258, 0.7160)	1.0566
Invasive type: plant	0.3109 (-0.4907, 1.1124)	0.4090
Year group: 2000-2010	-0.7087 (-2.1323, 0.7150)	0.7264
Year group: 2011-2020	-0.5906 (-1.9786, 0.7975)	0.7082
Year group: post-2020	-1.9965 (-3.8370, -0.1561)	0.9390
Biome: Tropical	1.1394 (-0.6618, 2.9405)	0.9190
Island or mainland: Mainland	0.8077 (-1.3393, 2.9547)	1.0954
Flight capability: Non-flying	-0.6811 (-1.2846, -0.0775)	0.3079

Supplementary Table 3. Study inclusion criteria. Inclusion criteria for studies assessing the impact of invasive alien species on native insect orders.

**Inclusion criterion**	**Description**
Focal insect orders	The study reports the impact of invasive alien species on native Hymenoptera, Coleoptera, Orthoptera, and/or Hemiptera.
Anthropogenic threat	The study focuses on invasive alien species (8.1 Invasive non-native/alien species/diseases) over other anthropogenic threats as defined by the IUCN threats classification scheme. For example, a study assessing the impact of an exotic tree plantation would be excluded as this is more closely aligned with the threat of land use change (e.g., 2.2 Wood & pulp plantations). Likewise, a study would be excluded if the effect of an invasive alien species was confounded by the effect of other threats.
Biodiversity metrics	The study reports measures of abundance, species richness, or biomass for the focal insect orders to at least taxonomic order level for a treatment (invasive alien species present) and a control (invasive alien species absent) site.
Temporal comparison	The treatment and control sites are compared at a given time, rather than historical records without the invasive alien species present being compared to current records with the invasive alien species present.
Experimental setting	Treatment and control sites are field sites either naturally existing or experimentally manipulated, for example through removing invasive alien species from control sites. Laboratory studies are excluded.
Invasive alien species	The invasive alien species is a single plant, animal, or disease.
Control site	The study ideally defines treatment and control sites as invasive alien present and invasive alien absent, respectively. However, studies where the invasive is not completely absent in the control site can be included if the study reports a measure of the invasive pressure (e.g., percentage cover).
Summary statistics	The study reports summary statistics for abundance, species richness, and biomass in treatment and control sites including mean, sample size, and a measure of variation. Alternatively, the study reports sufficient primary data to be able to calculate these values. The mean biodiversity measure for both treatment and control sites must be greater than zero (i.e., both have insects present).

Supplementary Note 1: Meta-analysis protocol

Title:

A meta-analysis of the impact of invasive species on multiple insect orders.

Objective of the meta-analysis (including the threat/order combinations, and PICO structured research question):

Threat = IUCN Red List Threat 8.1: Invasive non-native/alien species/diseases: Harmful plants, animals, pathogens and other microbes not originally found within the ecosystem(s) in question and directly or indirectly introduced and spread into it by human activities.

Orders = Hymenoptera, Coleoptera, Orthoptera, and Hemiptera (primarily terrestrial orders where invasive species are identified as a major potential threat from a previous expert elicitation process; Bladon *et al.*, 2023 unpublished)

PICO question = What is the impact of invasive species on the abundance, biomass, and species richness of the following orders: Hymenoptera, Coleoptera, Orthoptera, and Hemiptera relative to areas without invasive species present?

Definitions of each of the PICO components:

Population = Insect orders: Hymenoptera, Coleoptera, Orthoptera, Hemiptera. Global.

Intervention = Invasive species present.

Comparator = No invasive species present.

Outcome = Abundance, biomass, and species richness.

Search strategy:

Databases accessed = Scopus and Web of Science

Search options = Title, Abstract, Keywords (Scopus) / Topic (Web of Science equivalent)

Only including peer-reviewed studies

Date of your planned search:

Initial with proto search string – 23rd Feb 2023

Final with optimal search string – 3rd March 2023

Initial proto search string:

Scopus format (returned 871 results):

(TITLE-ABS-KEY({hymenoptera} OR {coleoptera} OR {orthoptera} OR {hemiptera}) AND TITLE-ABS-KEY({invasive} OR {alien}) AND TITLE-ABS-KEY({abundance} OR {biomass} OR {richness}))

Web of Science format (returned 1336 results):

{hymenoptera} OR {coleoptera} OR {orthoptera} OR {hemiptera} (Topic) AND {invasive} OR {alien} (Topic) AND {abundance} OR {biomass} OR {richness} (Topic)

Explanation as to how you will refine your search string:

- I will use my initial proto search string to search both Scopus and Web of Science databases.
- I will assess what proportion of the first 20 studies returned are relevant by reading their title and abstract to grasp how well the search string is performing at returning relevant results.
- Of the relevant papers, I will identify terms relevant to the research question that can subsequently be included in the search string to return further relevant results.
    - For example, I will include the terms: non-native, introduced, and exotic, in addition to the initial terms: invasive, and alien.
- I will use the litsearchr R package[4] to extract terms that commonly come up in tagged keywords so these can be added to the search string.
- I will stop adding search terms when the number of results returned leads to diminishing returns.
    - For example, not many papers were being returned for the Orthoptera insect order. Adding ‘grasshopper’ substantially increased the number of relevant papers returned, whereas adding ‘locust’ and/or ‘cricket’ did not.
- Of the irrelevant papers, I will identify the terms common to these that lead to them being returned in the search even though they are irrelevant to the research question. These exclusionary terms will be added to the search string.
    - For example, papers which only describe the distribution of invasive species can be excluded by adding the exclusionary terms: distribution, monitoring, and spread to the search string.

Bibliographic databases used:

Scopus and Web of Science

Inclusion criteria for each study:

1. Study should report the effect/impact of invasive species on populations of Hymenoptera, Coleoptera, Orthoptera, and/or Hemiptera.
2. Study should report measures of abundance, biomass, or species richness for Hymenoptera, Coleoptera, Orthoptera, and/or Hemiptera for a treatment (invasive species present) and a control (invasive species absent) area/plot.
    a. Treatment and control areas should be compared at a given time (rather than comparing historical records without an invasive species present to current records with the invasive species present).

b. Treatment and control areas can either be naturally existing or set up through experimental manipulation (e.g. through adding or removing invasive species to plots).
c. The invasive species needs to be a plant, animal, or disease. This should be recorded in the 'Treatment' column of the data extraction spreadsheet as "Invasive plant", "Invasive animal", or "Invasive disease".
d. Treatment and control areas are likely to be defined by the presence or absence of an invasive species. However, it will still be useful to include studies which compare areas with varying levels of invasive pressure. These studies can be included as long as the studies report a measure of the invasive pressure.

3. Study should report summary statistics for treatment and control groups including mean, sample size, and a measure of variation.
    a. Or report sufficient primary data to be able to calculate these values.

Screening strategy:

1. I will download bibliographic information (title, abstract, and citation information) for returned studies from Scopus and Web of Science, and remove duplicate studies using the litsearchr R package[4].
2. I will perform two screening rounds:
    a. 1st screening round: screen the titles and abstracts of the list of papers and discard all studies that are obviously irrelevant to the research question. Do this using the metagear R package[5].
    b. 2nd screening round: download the full text of each remaining study and perform a full-text screen. Exclude studies which don't meet the inclusion criteria and reason why (as this information is needed for the PRISMA diagram).

Data extraction approach:

See the standardised data extraction spreadsheet (https://osf.io/usj27) for details on the variables I aim to collect data for.

Data will be extracted from the main text or appendices/supplementary material of research papers. If presented as a table, data can be extracted directly. If the results are presented as figures, data will be extracted using the shinyDigitise R package[6].

Supplementary Note 2: Koricheva and Gurevitch (2014)[1] checklist

Criteria	Current study
1. Has formal meta-analysis been conducted (i.e. combination of effect sizes using standard meta-analytical methodology) or is it simply a vote count?	Yes. Methods sections details the methodology.
2. Are details of bibliographic search (electronic data bases used, keyword combinations, years) reported in sufficient detail to allow replication?	Yes. Protocol included as supplementary file contains this information. Also see PRISMA diagram included as supplementary fig. 4.
3. Are criteria for study inclusion/exclusion explicitly listed?	Yes. Criteria briefly described in methods section. Supplementary table 3 describes the Study inclusion criteria in detail.
4. Have standard metrics of effect size been used or, if non-standard metrics have been employed, is the distribution of these parameters known and have the authors explained how they calculated variances for such metrics?	Yes. Log response ratio used as effect size. LRR calculated with the metafor R package[2].
5. If more than one estimate of effect size per study was included in the analysis, has potential non-independence of these estimates been taken into account?	Yes, study identification and observation identification are included as nested random effects.
6. Have effect sizes been weighted by study precision or has the rational for using unweighted approach been provided?	Yes, the effect sizes are weighted by variance (via the metafor R package[2]).
7. Have statistical model for meta-analysis and the software used been described?	Yes. Methods sections details the methodology. Metafor R package[2] (rma.mv() function) used to run random-effects models.
8. Has heterogeneity of effect sizes between studies been quantified?	Yes. Methods sections details the methodology, and results section outlines results. Heterogeneity measure multi-level I^2 calculated and reported for abundance and species richness models. Confidence intervals

	and prediction intervals for overall effect size provided for each model.
9. Have the causes of existent heterogeneity in effect sizes been explored by meta-regression?	Yes. Moderators have been included to explore effect on heterogeneity.
10. If effects of multiple moderators have been tested, have potential non-independence of and interactions between moderators been taken into account?	Yes. Pearson correlation used to check for highly correlated moderators.
11. If meta-analysis combined studies conducted on different species, has phylogenetic relatedness of species been taken into account?	Yes. Models were run with 'order' as a moderator. We also explored whether incorporating a phylogeny matrix as a random effect improved fit of the abundance model.
12. Have tests for publication bias been conducted?	Yes. Funnel plots generated, rank correlation tests run, and an adapted version of Egger's regression run for each model.
13. If meta-analysis combines studies published over considerable time span, have possible temporal changes in effect size been tested?	Yes. Models were run with 'Year' as a moderator.
14. Have sensitivity analysis been performed to test the robustness of results?	Yes. Analysis repeated: 1) with Hedge's g as an alternative effect size, 2) without data points identified as particularly influential by Cook's distance, 3) without data collected with aquatic sampling techniques, 4) with only those data points that passed Geary's test, and 5) with incorporating a phylogenetic correlation matrix as a random effect.
15. Have full bibliographic details of primary studies included in a meta-analysis been provided?	Yes. See Supplementary Note 3.
16. Has the data set used for meta-analysis, including effect sizes and variances/sample sizes from individual primary studies and moderator variables, been provided as electronic appendix?	Yes. See the 'Data availability' statement in the main manuscript for access details.

<u>Supplementary Note 3: Citations for all studies included in the meta-analysis</u>

Altfeld, L., & Stiling, P. (2009). Effects of aphid-tending Argentine ants, nitrogen enrichment and early-season herbivory on insects hosted by a coastal shrub. Biological Invasions, 11(2), 183–191. https://doi.org/10.1007/s10530-008-9223-4
Berman, M., Andersen, A. N., & Ibanez, T. (2013). Invasive ants as back-seat drivers of native ant diversity decline in New Caledonia. Biological Invasions, 15(10), 2311–2331. https://doi.org/10.1007/s10530-013-0455-6
de Groot, M., Kleijn, D., & Jogan, N. (2007). Species groups occupying different trophic levels respond differently to the invasion of semi-natural vegetation by Solidago canadensis. Biological Conservation, 136(4), 612–617. https://doi.org/10.1016/j.biocon.2007.01.005
Devenish, A. J. M., Newton, R. J., Bridle, J. R., Gomez, C., Midgley, J. J., & Sumner, S. (2021). Contrasting responses of native ant communities to invasion by an ant invader, Linepithema humile. Biological Invasions, 23(8), 2553–2571. https://doi.org/10.1007/s10530-021-02522-7
Elleriis, P., Pedersen, M. L., & Toft, S. (2015). Impact of invasive Rosa rugosa on the arthropod fauna of Danish yellow dunes. Biological Invasions, 17(11), 3289–3302. https://doi.org/10.1007/s10530-015-0953-9
Ellis, L. M., Molles, M. C., Crawford, C. S., & Heinzelmann, F. (2000). Surface-Active Arthropod Communities in Native and Exotic Riparian Vegetation in the Middle Rio Grande Valley, New Mexico. The Southwestern Naturalist, 45(4), 456–471. https://doi.org/10.2307/3672594
Emery, S. M., & Doran, P. J. (2013). Presence and management of the invasive plant Gypsophila paniculata (baby’s breath) on sand dunes alters arthropod abundance and community structure. Biological Conservation, 161, 174–181. https://doi.org/10.1016/j.biocon.2013.03.015
Epperson, D. M., & Allen, C. R. (2010). Red Imported Fire Ant Impacts on Upland Arthropods in Southern Mississippi. The American Midland Naturalist, 163(1), 54–63. https://doi.org/10.1674/0003-0031-163.1.54
Ernsting, G., Block, W., MacAlister, H., & Todd, C. (1995). The invasion of the carnivorous carabid beetle Trechisibus antarcticus on South Georgia (sub-Antarctic) and its effect on the endemic herbivorous beetle Hydromedion spasutum. Oecologia, 103(1), 34–42. https://doi.org/10.1007/BF00328422
Estany-Tigerström, D., Bas, J. M., & Pons, P. (2010). Does Argentine ant invasion affect prey availability for foliage-gleaning birds? Biological Invasions, 12(4), 827–839. https://doi.org/10.1007/s10530-009-9504-6
Flanders, A. A., Kuvlesky, W. P., Jr., Ruthven, D. C., III, Zaiglin, R. E., Bingham, R. L., Fulbright, T. E., Hernández, F., & Brennan, L. A. (2006). Effects of Invasive Exotic Grasses on South Texas Rangeland Breeding Birds. The Auk, 123(1), 171–182. https://doi.org/10.1093/auk/123.1.171
Freeland-Riggert, B. T., Cairns, S. H., Poulton, B. C., & Riggert, C. M. (2016). Differences Found in the Macroinvertebrate Community Composition in the Presence or Absence of the Invasive Alien Crayfish, Orconectes hylas. PLOS ONE, 11(3), e0150199. https://doi.org/10.1371/journal.pone.0150199

Gippet, J. M. W., George, L., & Bertelsmeier, C. (2022). Local coexistence of native and invasive ant species is associated with micro-spatial shifts in foraging activity. Biological Invasions, 24(3), 761–773. https://doi.org/10.1007/s10530-021-02678-2

Goodman, M., & Warren II, R. J. (2019). Non-native ant invader displaces native ants but facilitates non-predatory invertebrates. Biological Invasions, 21(8), 2713–2722. https://doi.org/10.1007/s10530-019-02005-w

Goulson, D., Stout, J. C., & Kells, A. R. (2002). Do exotic bumblebees and honeybees compete with native flower-visiting insects in Tasmania? Journal of Insect Conservation, 6(3), 179–189. https://doi.org/10.1023/A:1023239221447

Hansen, A. K., Ortega, Y. K., & Six, D. L. (2009). Comparison of Ground Beetle (Coleoptera: Carabidae) Assemblages in Rocky Mountain Savannas Invaded and Un-Invaded by an Exotic Forb, Spotted Knapweed. Northwest Science, 83(4), 348–360. https://doi.org/10.3955/046.083.0406

Harris, R., Toft, R., Dugdale, J., Williams, P. A., & Rees, J. (2004). Insect assemblages in a native (kanuka—Kunzea ericoides) and an invasive (gorse—Ulex europaeus) shrubland. New Zealand Journal of Ecology, 28(1), 35–47.

Hasin, S., Tasen, W., Ohashi, M., Boonriam, W., & Yamada, A. (2021). Yellow crazy ants (Anoplolepis gracilipes [Smith, F., 1857]: Hymenoptera: Formicidae) threaten community of ground-dwelling arthropods in dry evergreen forests of Thailand. Agriculture and Natural Resources, 55, 634–643. https://doi.org/10.34044/j.anres.2021.55.4.14

Holway, D. A. (1998). Effect of Argentine ant invasions on ground-dwelling arthropods in northern California riparian woodlands. Oecologia, 116(1), 252–258. https://doi.org/10.1007/s004420050586

Houston, W. A., & Duivenvoorden, L. J. (2002). Replacement of littoral native vegetation with the ponded pasture grass Hymenachne amplexicaulis: Effects on plant, macroinvertebrate and fish biodiversity of backwaters in the Fitzroy River, Central Queensland, Australia. Marine and Freshwater Research, 53(8), 1235–1244. https://doi.org/10.1071/mf01042

Huang, S.-C., Norval, G., Wei, C.-S., & Tso, I.-M. (2008). Effects of the brown anole invasion and betelnut palm planting on arthropod diversity in southern Taiwan. Zoological Science, 25(11), 1121–1129. https://doi.org/10.2108/zsj.25.1121

Ito, H. C., Shiraishi, H., Nakagawa, M., & Takamura, N. (2020). Combined impact of pesticides and other environmental stressors on animal diversity in irrigation ponds. PLOS ONE, 15(7), e0229052. https://doi.org/10.1371/journal.pone.0229052

Jochum, M., Thouvenot, L., Ferlian, O., Zeiss, R., Klarner, B., Pruschitzki, U., Johnson, E. A., & Eisenhauer, N. (2022). Aboveground impacts of a belowground invader: How invasive earthworms alter aboveground arthropod communities in a northern North American forest. Biology Letters, 18(3), 20210636. https://doi.org/10.1098/rsbl.2021.0636

King, J. R., & Tschinkel, W. R. (2006). Experimental evidence that the introduced fire ant, Solenopsis invicta, does not competitively suppress co-occurring ants in a disturbed habitat. Journal of Animal Ecology, 75(6), 1370–1378. https://doi.org/10.1111/j.1365-2656.2006.01161.x

Lester, P., & Tavite, A. (2004). Long-Legged Ants, Anoplolepis gracilipes (Hymenoptera: Formicidae), Have Invaded Tokelau, Changing Composition and Dynamics of Ant and Invertebrate Communities. Pacific Science, 58. https://doi.org/10.1353/psc.2004.0031

Loomis, J. D., & Cameron, G. N. (2014). Impact of the invasive shrub Amur honeysuckle (Lonicera maackii) on shrub-layer insects in a deciduous forest in the eastern United States. Biological Invasions, 16(1), 89–100. https://doi.org/10.1007/s10530-013-0505-0
Lopezaraiza–Mikel, M. E., Hayes, R. B., Whalley, M. R., & Memmott, J. (2007). The impact of an alien plant on a native plant–pollinator network: An experimental approach. Ecology Letters, 10(7), 539–550. https://doi.org/10.1111/j.1461-0248.2007.01055.x
Motard, E., Dusz, S., Geslin, B., Akpa-Vinceslas, M., Hignard, C., Babiar, O., Clair-Maczulajtys, D., & Michel-Salzat, A. (2015). How invasion by Ailanthus altissima transforms soil and litter communities in a temperate forest ecosystem. Biological Invasions, 17(6), 1817–1832. https://doi.org/10.1007/s10530-014-0838-3
Natsumeda, T., Takamura, N., Nakagawa, M., Kadono, Y., Tanaka, T., & Mitsuhashi, H. (2015). Environmental and biotic characteristics to discriminate farm ponds with and without exotic largemouth bass and bluegill in western Japan. Limnology, 16(3), 139–148. https://doi.org/10.1007/s10201-015-0453-8
Nguyen, K. Q., Cuneo, P., Cunningham, S. A., Krix, D. W., Leigh, A., & Murray, B. R. (2016). Ecological effects of increasing time since invasion by the exotic African olive (Olea europaea ssp. Cuspidata) on leaf-litter invertebrate assemblages. Biological Invasions, 18(6), 1689–1699. https://doi.org/10.1007/s10530-016-1111-8
Ogura-Yamada, C. S., & Krushelnycky, P. D. (2020). The effects of the invasive thief ant, Solenopsis papuana, on ground-dwelling invertebrates in mesic forests of Hawaiʻi. Journal of Insect Conservation, 24(1), 151–162. https://doi.org/10.1007/s10841-019-00185-3
Ortega, Y. K., McKelvey, K. S., & Six, D. L. (2006). Invasion of an exotic forb impacts reproductive success and site fidelity of a migratory songbird. Oecologia, 149(2), 340–351. https://doi.org/10.1007/s00442-006-0438-8
Osbrink, W. L. A., Thomas, D. B., Goolsby, J. A., Showler, A. T., & Leal, B. (2018). Higher Beetle Diversity in Native Vegetation Than in Stands of the Invasive Arundo, Arundo donax L., along the Rio Grande Basin in Texas, USA. Journal of Insect Science, 18(3), 18. https://doi.org/10.1093/jisesa/iey053
Ramula, S., & Sorvari, J. (2017). The invasive herb Lupinus polyphyllus attracts bumblebees but reduces total arthropod abundance. Arthropod-Plant Interactions, 11(6), 911–918. https://doi.org/10.1007/s11829-017-9547-z
Růžičková, J., & Hykel, M. (2019). Habitat mosaic of gravel pit as a potential refuge for carabids: A case study from Central Europe. Community Ecology, 20(3), 215–222. https://doi.org/10.1556/168.2019.20.3.1
Sahli, H. F., Krushelnycky, P. D., Drake, D. R., & Taylor, A. D. (2016). Patterns of floral visitation to native Hawaiian plants in presence and absence of invasive Argentine ants. Pacific Science, 70(3), 309–323. https://doi.org/10.2984/70.3.3
Salyer, A., Bennett, G. W., & Buczkowski, G. A. (2014). Odorous House Ants (Tapinoma sessile) as Back-Seat Drivers of Localized Ant Decline in Urban Habitats. PLOS ONE, 9(12), e113878. https://doi.org/10.1371/journal.pone.0113878
Sarty, M., Abbott, K. L., & Lester, P. J. (2007). Community level impacts of an ant invader and food mediated coexistence. Insectes Sociaux, 54(2), 166–173. https://doi.org/10.1007/s00040-007-0927-8
Sasal, Y., Raffaele, E., & Farji-Brener, A. G. (2015). Consequences of fire and cattle browsing on ground beetles (Coleoptera) in NW Patagonia. Ecological Research, 30(6), 1015–1023. https://doi.org/10.1007/s11284-015-1302-2

Savage, A. M., Rudgers, J. A., & Whitney, K. D. (2009). Elevated dominance of extrafloral nectary-bearing plants is associated with increased abundances of an invasive ant and reduced native ant richness. Diversity and Distributions, 15(5), 751–761. https://doi.org/10.1111/j.1472-4642.2009.00579.x
Schirmel, J., Timler, L., & Buchholz, S. (2011). Impact of the invasive moss Campylopus introflexus on carabid beetles (Coleoptera: Carabidae) and spiders (Araneae) in acidic coastal dunes at the southern Baltic Sea. Biological Invasions, 13(3), 605–620. https://doi.org/10.1007/s10530-010-9852-2
Schuh, M., & Larsen, K. J. (2015). Rhamnus cathartica (Rosales: Rhamnaceae) Invasion Reduces Ground-Dwelling Insect Abundance and Diversity in Northeast Iowa Forests. Environmental Entomology, 44(3), 647–657. https://doi.org/10.1093/ee/nvv050
Stanley, M. C., Nathan, H. W., Phillips, L. K., Knight, S. J., Galbraith, J. A., Winks, C. J., & Ward, D. F. (2013). Invasive interactions: Can Argentine ants indirectly increase the reproductive output of a weed? Arthropod-Plant Interactions, 7(1), 59–67. https://doi.org/10.1007/s11829-012-9215-2
Stanley, M. C., & Ward, D. F. (2012). Impacts of Argentine ants on invertebrate communities with below-ground consequences. Biodiversity and Conservation, 21(10), 2653–2669. https://doi.org/10.1007/s10531-012-0324-0
Staubus, W. J., Boyd, E. S., Adams, T. A., Spear, D. M., Dipman, M. M., & Meyer, W. M. (2015). Ant communities in native sage scrub, non-native grassland, and suburban habitats in Los Angeles County, USA: Conservation implications. Journal of Insect Conservation, 19(4), 669–680. https://doi.org/10.1007/s10841-015-9790-5
Tanner, R. A., Varia, S., Eschen, R., Wood, S., Murphy, S. T., & Gange, A. C. (2013). Impacts of an Invasive Non-Native Annual Weed, Impatiens glandulifera, on Above- and Below-Ground Invertebrate Communities in the United Kingdom. PLOS ONE, 8(6), e67271. https://doi.org/10.1371/journal.pone.0067271
Vonshak, M., Dayan, T., Ionescu-Hirsh, A., Freidberg, A., & Hefetz, A. (2010). The little fire ant Wasmanniaauropunctata: A new invasive species in the Middle East and its impact on the local arthropod fauna. Biological Invasions, 12(6), 1825–1837. https://doi.org/10.1007/s10530-009-9593-2
Walker, K. L. (2006). Impact of the Little Fire Ant, Wasmannia auropunctata, on Native Forest Ants in Gabon. Biotropica, 38(5), 666–673. https://doi.org/10.1111/j.1744-7429.2006.00198.x
Walters, A. C. (2006). Invasion of Argentine ants (Hymenoptera: Formicidae) in South Australia: Impacts on community composition and abundance of invertebrates in urban parklands. Austral Ecology, 31(5), 567–576. https://doi.org/10.1111/j.1442-9993.2006.01592.x
Watts, C., Rohan, M., & Thornburrow, D. (2012). Beetle community responses to grey willow (Salix cinerea) invasion within three New Zealand wetlands. New Zealand Journal of Zoology, 39(3), 209–227. https://doi.org/10.1080/03014223.2011.645838
Weyl, P. S., de Moor, F. C., Hill, M. P., & Weyl, O. L. (2010). The effect of largemouth bass Micropterus salmoides on aquatic macro-invertebrate communities in the Wit River, Eastern Cape, South Africa. African Journal of Aquatic Science, 35(3), 273–281. https://doi.org/10.2989/16085914.2010.540776

Yoshioka, A., Kadoya, T., Suda, S., & Washitani, I. (2010). Invasion of weeping lovegrass reduces native food and habitat resource of Eusphingonotus japonicus (Saussure). Biological Invasions, 12(8), 2789–2796. https://doi.org/10.1007/s10530-009-9684-0

Supplementary References

1. Koricheva, J. & Gurevitch, J. Uses and misuses of meta-analysis in plant ecology. *Journal of Ecology* **102**, 828–844 (2014).
2. Viechtbauer, W. Conducting meta-analyses in R with the metafor package. *Journal of statistical software* **36**, 1–48 (2010).
3. Lajeunesse, M. J. Bias and correction for the log response ratio in ecological meta-analysis. *Ecology* **96**, 2056–2063 (2015).
4. Grames, E. M., Stillman, A. N., Tingley, M. W. & Elphick, C. S. An automated approach to identifying search terms for systematic reviews using keyword co-occurrence networks. *Methods in Ecology and Evolution* **10**, 1645–1654 (2019).
5. Lajeunesse, M. J. metagear: Comprehensive Research Synthesis Tools for Systematic Reviews and Meta-Analysis. (2021).
6. Ivimey-Cook, E. R., Noble, D. W. A., Nakagawa, S., Lajeunesse, M. J. & Pick, J. L. Advice for improving the reproducibility of data extraction in meta-analysis. *Research Synthesis Methods* **14**, 911–915 (2023).
