# Supplementary material for: Meta-analysis reveals negative but highly variable impacts of invasive alien species across terrestrial insect orders
Source: Nat Commun. 2026 Jan 15;17:296. doi: 10.1038/s41467-025-67925-9 (PMC12808215; doi:10.1038/s41467-025-67925-9)
Supplement: Supplementary file 2 — Description of Additional Supplementary Information [file 41467_2025_67925_MOESM2_ESM.pdf]

Description of Additional Supplementary Files

File Name: Supplementary Data 1
Description: **Study screening outcomes and exclusion reasons**. Excel spreadsheet presenting the screening and exclusion information for all studies considered for inclusion in the meta analysis. The file contains three tabs:
1-title & abstract screen: Inclusion outcomes for every study screened during the first screening round (title and abstract screening).
2-exclusion at full-text screen: Exclusion reasons for each study removed during the second screening round (full-text screening).
3-exclusion at data extraction: Exclusion reasons for each study removed during the data extraction stage.
