# Supplementary material for: Meta-analysis reveals negative but highly variable impacts of invasive alien species across terrestrial insect orders
Source: Nat Commun. 2026 Jan 15;17:296. doi: 10.1038/s41467-025-67925-9 (PMC12808215; doi:10.1038/s41467-025-67925-9)
Supplement: Supplementary file 4 — Reporting Summary [file 41467_2025_67925_MOESM4_ESM.pdf]

nature portfolio

Corresponding author(s): Grace Skinner

Last updated by author(s): 18/11/25

Reporting Summary

Nature Portfolio wishes to improve the reproducibility of the work that we publish. This form provides structure for consistency and transparency in reporting. For further information on Nature Portfolio policies, see our Editorial Policies and the Editorial Policy Checklist.

Statistics

For all statistical analyses, confirm that the following items are present in the figure legend, table legend, main text, or Methods section.

n/a	Confirmed	
☐	☒	The exact sample size (*n*) for each experimental group/condition, given as a discrete number and unit of measurement
☐	☒	A statement on whether measurements were taken from distinct samples or whether the same sample was measured repeatedly
☐	☒	The statistical test(s) used AND whether they are one- or two-sided<br>*Only common tests should be described solely by name; describe more complex techniques in the Methods section.*
☐	☒	A description of all covariates tested
☐	☒	A description of any assumptions or corrections, such as tests of normality and adjustment for multiple comparisons
☐	☒	A full description of the statistical parameters including central tendency (e.g. means) or other basic estimates (e.g. regression coefficient) AND variation (e.g. standard deviation) or associated estimates of uncertainty (e.g. confidence intervals)
☒	☐	For null hypothesis testing, the test statistic (e.g. *F*, *t*, *r*) with confidence intervals, effect sizes, degrees of freedom and *P* value noted<br>*Give P values as exact values whenever suitable.*
☒	☐	For Bayesian analysis, information on the choice of priors and Markov chain Monte Carlo settings
☒	☐	For hierarchical and complex designs, identification of the appropriate level for tests and full reporting of outcomes
☐	☒	Estimates of effect sizes (e.g. Cohen's *d*, Pearson's *r*), indicating how they were calculated

Our web collection on statistics for biologists contains articles on many of the points above.

Software and code

Policy information about availability of computer code

Data collection	No software was used for data collection.
Data analysis	The primary code for data analysis was written in R version 4.4.1. The code written to perform the data analyses is available on Zenodo [https://zenodo.org/records/15611484; DOI: 10.5281/zenodo.14290020]. All R packages used to perform the analyses are listed within the code, and where applicable packages are referenced within the main text. The R packages litsearchr 1.0.0, metagear 0.7, and shinyDigitise 0.1.0 were used to identify studies which met our inclusion criteria for the meta-analysis, and for extracting data from figures. The following package list includes the remaining packages used for data analysis and visualisation: writexl 1.5.1, metafor 4.6-0, dmetar 0.1.0, tidyverse 2.0.0, rnaturalearth 1.0.1, waffle 1.0.2, ggimage 0.3.3, orchaRd 2.0, rotl 3.1.0, ape 5.8-1, stringr 1.5.1, Polychrome 1.5.4, and cowplot 1.1.3.

For manuscripts utilizing custom algorithms or software that are central to the research but not yet described in published literature, software must be made available to editors and reviewers. We strongly encourage code deposition in a community repository (e.g. GitHub). See the Nature Portfolio guidelines for submitting code & software for further information.

Data

Policy information about availability of data

All manuscripts must include a data availability statement. This statement should provide the following information, where applicable:

- Accession codes, unique identifiers, or web links for publicly available datasets
- A description of any restrictions on data availability
- For clinical datasets or third party data, please ensure that the statement adheres to our policy

The data generated and analysed in this study have been deposited in the Zenodo repository at https://zenodo.org/records/15611484 (DOI: 10.5281/zenodo.14290020). Data from one contributing study are subject to data-sharing restrictions imposed by the data provider and therefore cannot be made publicly available. Access to these data can be obtained by contacting the original data owner. Analyses using the shared dataset reproduce the reported results with only minor quantitative differences due to this omission. Supplementary Note 3 provides a complete list of references for all studies from which data were extracted for inclusion in the meta-analysis.

Research involving human participants, their data, or biological material

Policy information about studies with human participants or human data. See also policy information about sex, gender (identity/presentation), and sexual orientation and race, ethnicity and racism.

Reporting on sex and gender	NA
Reporting on race, ethnicity, or other socially relevant groupings	NA
Population characteristics	NA
Recruitment	NA
Ethics oversight	NA

Note that full information on the approval of the study protocol must also be provided in the manuscript.

Field-specific reporting

Please select the one below that is the best fit for your research. If you are not sure, read the appropriate sections before making your selection.

☐ Life sciences ☐ Behavioural & social sciences ☒ Ecological, evolutionary & environmental sciences

For a reference copy of the document with all sections, see nature.com/documents/nr-reporting-summary-flat.pdf

Ecological, evolutionary & environmental sciences study design

All studies must disclose on these points even when the disclosure is negative.

Study description	We compiled existing data to complete a meta-analysis assessing the impact of invasive alien species on insects (Coleoptera, Hemiptera, Hymenoptera, and Orthoptera), and examining factors influencing these effects. No primary investigation was undertaken, instead we extracted and collated existing data from 52 primary studies, which met our inclusion criteria.
Research sample	Following the PRISMA methodology, we collated studies assessing the impact of invasive alien species on the abundance, biomass, and species richness of our focal taxa (i.e., insects in the orders Hymenoptera, Coleoptera, Orthoptera, and Hemiptera), relative to areas without invasive alien species present. We used a search string to search Scopus and Web of Science databases to return peer-reviewed, primary research studies that might be relevant to our question. We did not impose a publication date cutoff. We then assessed each study against our inclusion criteria detailed in the manuscript. Finally, we extracted data describing pairwise comparisons of the abundance, species richness, and biomass of insects in sites with and without invasive alien species present.
Sampling strategy	Our meta-analysis was comprehensive such that all known relevant primary studies and their data points have been included.
Data collection	318 data points were extracted from 52 primary studies. Citations for all studies included in the meta-analysis are provided by Supplementary Note 3 in the Supplementary Information file.
Timing and spatial scale	We did not impose a publication date cutoff or geographical restriction on the compiled studies/data. Date of publication of included studies ranged from 1995 to 2022. Data is included from every continent except Antarctica.
Data exclusions	We extracted data from 53 studies that met our inclusion criteria for our meta-analysis. For one of these studies, the researchers measured the biomass of insects, whereas the remaining 52 studies measured abundance or species richness. These three biomass data points were excluded from further analysis as the models were run separately for each biodiversity metric, and there were too few data points for biomass. One further effect size was excluded from further analysis because it had substantially greater variance than all others (more than 25 times greater adjusted variance than the effect size with the second greatest adjusted variance), due to a relatively large standard deviation on a mean that was less than 0.1 (i.e., a poorly sampled insect species). This high variance effect

size was removed before running any meta-analytic models.

Reproducibility	No experimental procedures undertaken. Analytical reproducibility is underpinned by the R analysis code available on Zenodo [https://zenodo.org/records/15611484 DOI: 10.5281/zenodo.14290020].
Randomization	As a meta-analysis, randomization is not applicable to our study, as we have synthesized data from previously conducted studies where we had no control over the original study designs.
Blinding	Blinding is not relevant to process of conducting a meta-analysis.

Did the study involve field work? ☐ Yes ☒ No

Reporting for specific materials, systems and methods

We require information from authors about some types of materials, experimental systems and methods used in many studies. Here, indicate whether each material, system or method listed is relevant to your study. If you are not sure if a list item applies to your research, read the appropriate section before selecting a response.

Materials & experimental systems

n/a	Involved in the study	
☒	☐	Antibodies
☒	☐	Eukaryotic cell lines
☒	☐	Palaeontology and archaeology
☒	☐	Animals and other organisms
☒	☐	Clinical data
☒	☐	Dual use research of concern
☒	☐	Plants

Methods

n/a	Involved in the study	
☒	☐	ChIP-seq
☒	☐	Flow cytometry
☒	☐	MRI-based neuroimaging

Plants

Seed stocks	*Report on the source of all seed stocks or other plant material used. If applicable, state the seed stock centre and catalogue number. If plant specimens were collected from the field, describe the collection location, date and sampling procedures.*
Novel plant genotypes	*Describe the methods by which all novel plant genotypes were produced. This includes those generated by transgenic approaches, gene editing, chemical/radiation-based mutagenesis and hybridization. For transgenic lines, describe the transformation method, the number of independent lines analyzed and the generation upon which experiments were performed. For gene-edited lines, describe the editor used, the endogenous sequence targeted for editing, the targeting guide RNA sequence (if applicable) and how the editor was applied.*
Authentication	*Describe any authentication procedures for each seed stock used or novel genotype generated. Describe any experiments used to assess the effect of a mutation and, where applicable, how potential secondary effects (e.g. second site T-DNA insertions, mosiacism, off-target gene editing) were examined.*
